# Demographic and service-use profiles of individuals using the CarePayment program for hospital-related medical debt: results from a nationwide survey of guarantors

**DOI:** 10.1186/s12913-016-1525-0

**Published:** 2016-07-15

**Authors:** Laura Lessard, Julie Solomon

**Affiliations:** University of Delaware, 017 Carpenter Sports Building, Newark, 19716 DE USA; J. Solomon Consulting, LLC, Mountain View, CA USA

**Keywords:** Medical bills, Hospital financing, Patient credit and financing, Debt

## Abstract

**Background:**

Many Americans find themselves with problems paying medical bills, and medical debt can lead to numerous negative financial, social and access to healthcare outcomes. One potential market-based solution to these challenges is to provide financing options that have patient-friendly terms while complying with increasingly complex federal lending regulations. CarePayment (CP) is one entity that provides zero interest financing to individuals from participating medical facilities. An independent, initial outcome study was undertaken to understand the demographic and medical debt-related outcomes of CP users. This information is integral to understanding whether and how this program can ameliorate the negative consequences of medical debt.

**Methods:**

A nationwide telephone survey was conducted with a random sample of 8122 guarantors who were paying off CarePayment debt as of January 1, 2015. Respondents were asked about their demographic characteristics as well as self-report of negative outcomes typically associated with medical debt. Analyses included descriptive statistics along with logistic regression models comparing first-time CP users and those with higher amounts of CP debt to others.

**Results:**

The most commonly reported financial challenge related to medical bills was problems paying or being unable to pay medical bills (59.5 %). The most commonly reported access-to-care challenges were skipping a medical test or treatment recommended by a doctor (32.9 %) and having a medical problem but not going to the doctor/clinic (30.3 %). Comparisons between first-time and repeat CP users suggest that first-time users were significantly more likely to report several negative outcomes and those with both CP and non-CP debt were significantly more likely to report nearly all of the undesirable financial and access outcomes that were assessed compared to those with only CP debt.

**Conclusions:**

The results suggest that CP use, especially repeat CP use, may be associated with a reduction in many negative outcomes of medical debt. In addition, while we found that individuals with only CP debt fared better than those with both CP debt and other medical debt, 60 % of our sample had more than one source of medical debt. This suggests that the beneficial impact of CP could be increased by expanding access to the program.

## Background

Americans commonly experience problems paying medical bills, and medical debt can cause numerous negative financial and healthcare access outcomes. The national Commonwealth Fund Biennial Health Insurance Survey (BHIS) documented a significant increase in medical bill debt and medical bill problems (defined as problems paying or inability to pay medical bills, being contacted by a collection agency about unpaid medical bills, and/or having to change one’s way of life in order to pay medical bills), during the 2000s. Specifically, in 2005, 34 % of adults ages 19–64 reported that within the past 12 months they had had some type of medical bill problem and/or medical debt. The rate of medical bill problems and/or debt rose significantly to 40 % in the 2010 BHIS; was statistically unchanged at 41 % in the 2012 BHIS; and declined significantly to 35 % in the 2014 BHIS [1]. The recent modest decline is likely due to implementation of the Affordable Care Act (ACA), which expanded health insurance coverage to many across the country [1]. Populations that are more likely to report medical bill problems and medical debt include the uninsured [2] and lower- and middle-income families [1]. In the 2014 BHIS, approximately two-thirds of those with medical debt owed less than $4000 [1]; however, the literature suggests that even small amounts of debt can lead to problems, and that medical bill problems can increase dramatically with amount of debt [3].

Individuals in families with medical bill problems commonly report at least one negative financial consequence, including problems paying for necessities like food, clothing and/or housing; using up savings; taking on credit card debt; receiving a lower credit rating; declaring bankruptcy or delaying career or education plans [4]. These outcomes may be the ones readily associated with medical debt, but in fact the impact of medical debt is also felt on individuals’ and families’ access to health care. Analyses of Michigan Recession and Recovery Study survey data, collected from southeastern Michigan residents in the wake of the late 2000s recession, showed that overall debt was positively associated with foregoing medical or dental care in the past 12 months, even after adjusting for socioeconomic and health characteristics, household income, and net worth. In particular, the associations between debt and forgoing care were shown to be driven largely by medical and credit card debt [5]. These negative financial and health care access consequences of medical debt can lead in turn to psychological stress, worsening health status, decreased ability to work, denial of employment (due to debt-related credit problems), wage withholding, and housing problems, such as inability to qualify for a mortgage, being turned down from renting a home, inability to make rent or mortgage payments, being forced to move to less expensive housing, and being evicted or made homeless [6–8].

Reducing the negative consequences of debt is a challenge that will likely require a multitude of solutions, including some that were included in recent 501c (r) revisions. Specifically, recent changes to the tax code require non-profit hospitals to a) adopt written financial assistance plans (FAP); and b) make “reasonable efforts to determine whether an individual is eligible for assistance under the hospital’s [FAP] before engaging in extraordinary actions against the individual” [9]. These rules and others are designed to increase transparency and improve access to charity care and other payment options available to patients and families. However, individuals are responsible for an increasing amount of debt due to both high co-payments and the dramatic increase in high-deductible health insurance plans [10]. A recent industry brief suggests that the source of unpaid hospital debt will shift from majority self-pay (primarily uninsured individuals) to majority balance after insurance (insured individuals) in the next five years [11]. Furthermore, some estimate that healthcare providers collect only $0.18 to $0.34 on the dollar from individuals with high deductible plans [12]. For hospitals, decreases in revenue could potentially cut into charity care and community benefit. For patients and families, even modest amounts of debt can lead to negative financial and health access outcomes.

One market-based solution is providing access to payment plans that comply with federal lending regulations while offering terms that reduce some of the negative outcomes that commonly accompany them. CarePayment (http://www.carepayment.com; hereafter referred to as CP) is one entity that provides zero interest loans to individuals from participating medical facilities. While the specific terms vary from site to site, typically CP is offered to individuals as a 25 month payment plan with monthly payments starting at 4 % of the debt amount, or $25, whichever is higher. CP procedures comply with the Healthcare Financial Management Association’s *Patient Friendly Billing Project*, which promotes clear and concise information on patient bills and statements [13].

We undertook an initial outcome study to document the demographic and CP service use profiles and medical debt-related outcomes of a nationally representative sample of CP guarantors. This information can be helpful in understanding who avails themselves of CP, along with the potential impact of CP on the negative outcomes described above. Other hospitals and medical facilities that are currently not using CP might consider adding CP or a similar type of service to their revenue cycle operations.

## Methods

### Sample & recruitment

In January 2015, in collaboration with CP, a random sample of 8122 CP guarantors was created from CP’s national database. The sample included guarantors from CP’s hospital clients, age 18 or older, who were paying off CP debt as of January 1, 2015. The principal reason for focusing on guarantors (who are usually also the CP patients), and not the patients per se, is that patients may be under the legal age of consent or be legally unable to consent to study participation due to diminished mental capacity. Guarantors are by definition of legal age and capacity to consent to participate. Some hospital clients (and their guarantors) were excluded from the sample due to restrictions in place within their Business Associates Agreements (BAA) with CP. CP provided only the names, addresses and telephone numbers of potential participants; other demographic information on the whole sample was not available. All members of the sample were sent an initial recruitment letter by mail which described the study’s aims and alerted potential participants to the study procedures. Pacific Market Research (PMR), an independent market research firm located in Washington State, was given a subcontract to conduct the telephone surveys.

After the recruitment letters were sent out, PMR made up to four calls to each potential participant over the course of approximately three weeks. The call center verified that the potential participant was making payments to CP, was of legal age to participate and could communicate in English. Guarantors who could not communicate in English were excluded. The desired sample size for the project (1000 respondents) was based on a power calculation designed to represent the approximately 40,600 guarantors who met inclusion criteria. The desired sample size drove the recruitment strategy. PMR made telephone calls to each potential participant until the 1000 respondent threshold was met; in the end 8075 potential participants were called at least once. Due to legal concerns related to the Telephone Consumer Protection Act, no voicemail messages were left for potential participants.

### Instrument

Once the potential participant consented to the research, the interviewer posed a series of questions about their medical debt and potential outcomes that could be attributed to medical debt, including both financial challenges (e.g., taking on credit card debt) and access to care (e.g., skipping doses of prescription medicine due to cost). The majority of the questions were drawn from the 2012 Biennial Health Insurance Survey (BHIS), and others were drawn from Kaiser Family Foundation surveys on health insurance and the Affordable Care Act. The survey also contained a number of demographic items designed to capture the profiles of guarantors, and questions about satisfaction with CP drawn from a previous satisfaction survey conducted by a CP consultant. At the conclusion of the survey, participants were given the option of receiving a $10 check via mail as a thank you for participation.

### Data analysis

A raw data file was created by PMR with the responses to each question. The file did not contain any identifying information about individual respondents (e.g., name, address, telephone number) to comply with HIPAA regulations. CP provided the total amount of debt the guarantor was currently paying off (high balance); this variable was added to the dataset using a unique identifier that linked the data file to CP’s database. The data analysis included simple descriptive statistics (frequencies, means) for all variables and cross-tabulations to compare categorical variables. Tables present sample n’s and valid percentages; respondents omitting answers to a particular question were excluded item-by-item from analyses.

Given the lack of comparison group in this study, the analyses also explored whether individuals using CP for the first time and individuals with other (non-CP) medical debt reported more negative outcomes compared to other guarantors. These analyses assumed that if CP participation is associated with reductions in negative outcomes associated with medical debt in the literature, then an argument can be made that CP influences these outcomes in a positive way. Logistic regression models were run with each of seventeen measured negative outcomes (e.g., taking on credit card debt, skipping needed medical treatments) as an outcome variable (yes/no) and indicator variables for these attributes as predictors. All seventeen models controlled for total amount of medical debt, which included a combination of CP debt and any other medical debt currently being paid off over time (categorical: <$2000; $2000–3999; $4000–7999; $8000–9999; $10,000 or more), along with household income (categorical: <$20,000; $20,000–39,999; $40,000–59,999; $60,000–79,999; $80,000 or more).

We received a Waiver of Prior Authorization under HIPAA in order to contact guarantors, and all study procedures were reviewed and approved by the Institutional Review Board of Arcadia University (Federal-wide assurance #00000449). While the researchers collaborated with CP in order to access information about guarantors (e.g., phone numbers, names, amount of debt), the study was conducted independently, such that CP staff were not involved in data collection, analysis or report writing. The study was funded by the W.K. Kellogg Foundation.

## Results

Ultimately, there were 1000 completed interviews included in the final data set (overall response rate: 12.4 %). The response rate was lower than other similar surveys due to the quota sampling used; some potential participants were only called once (*n* = 2126; 26.3 %) or twice (*n* = 1431; 17.8 %).

### Demographics & service use profile

The first research question focused on understanding the demographic and service-use profiles of guarantors. This was important because neither CP nor the client hospitals collected or maintained records on demographic characteristics of guarantors. Table 1, below, presents the results for each relevant survey item. The majority of the guarantors were non-Hispanic/Latino (93.9 %) and White (88.6 %). Over one third of the sample (35.5 %) reported their highest education level to be high school or equivalent, and a smaller number were college graduates (20.0 %) or higher (7.1 %). While 63.6 % of respondents reported being married, 60.6 % reported having no dependent children age 23 or younger. This may be explained by the higher age distribution of our sample; the average age of guarantors was 52 years (data not shown).

**Table 1:** Demographic and Service-Use Profiles of Guarantors

	Respondents	
	# respondents	% total^a^
Demographic Characteristics		
Ethnicity (*n* = 989)		
Not Hispanic or Latino	929	93.9
Hispanic or Latino	60	6.1
Race (*n* = 979)		
White	867	88.6
African-American	48	4.9
Multiracial	26	2.7
Other (including Asian)	38	3.9
Education Level (*n* = 994)		
Less than high school	62	6.2
High school graduate or equivalent	353	35.5
Some college but no degree	309	31.1
College graduate	199	20.0
Postgraduate	71	7.1
Marital Status (*n* = 994)		
Married	632	63.6
Living with partner	38	3.8
Divorced	156	15.7
Separated	12	1.2
Widowed	78	7.8
Never married	78	7.8
Household Composition		
Dependent children 23 years of age or younger (*n* = 994)		
One child	142	14.3
More than one child	250	25.2
No children	602	60.6
Number of family members living in the home, including respondent (*n* = 995)		
One	188	18.9
Two	360	36.2
Three	175	17.6
Four or more	272	27.3
Employment Status (*n* = 998)		
Employed full-time	523	52.4
Employed part-time	91	9.1
Retired	223	22.3
Disabled	76	7.6
Other	85	8.5
Household Income (*n* = 856)		
Less than $20,000	153	17.9
$20,000–$39,999	260	30.4
$40,000–$59,999	225	26.3
$60,000–$79,999	114	13.3
$80,000–$99,999	60	7.0
$100,000 or more	44	5.1
Health Insurance status of patient at time of debt (*n* = 995)		
Private Insurance through an employer/union	673	67.6
Private Insurance purchased by guarantor	186	18.7
Medicare	236	23.7
Other	9	0.9
Uninsured at time of service	44	4.4
Service Use Profile		
Experience using CarePayment (*n* = 997)		
First time using CarePayment	714	71.6
Have used CarePayment before	272	27.3
Unsure	11	1.1
Amount of CarePayment Debt (*n* = 996)		
Less than $500	184	18.5
$500–$999	274	27.5
$1,000–$2,499	378	38.0
$2,500 or more	160	16.1
Other medical debt being paid off over time within past 12 months (*n* = 986)		
Yes	588	59.6
No	398	40.4
Approximate amount of other bills being paid off (*n* = 585)		
Less than $2,000	314	53.7
$2,000 to less than $4,000	130	22.2
$4,000 to less than $8,000	74	12.6
$8,000 to less than $10,000	16	2.7
$10,000 or more	25	4.3
Don’t know	26	4.4

^a^Percentages represent proportion of respondents that answered each question; percentages may not sum to 100 due to rounding

In terms of service usage, over 70 % of respondents reported that this was their first experience with CarePayment. The amount of debt financed using CP ranged from $42 to $18,601, with the majority (65.5 %) paying off between $500 and $2499. More than half of respondents (59.6 %) had additional (non-CP) medical bills they were paying off over time within the past 12 months, including those being paid with credit card, through personal loans or bill paying arrangements. Over half of these respondents (53.7 %) owed less than $2000 of such debt.

### Medical debt-related outcomes

Guarantors reported financial challenges they have faced within the past 12 months or two years (consistent with the timing and phrasing of BHIS items). The most commonly reported challenges were problems paying or being unable to pay medical bills (59.5 %), changing one’s way of life significantly in order to pay medical bills (38.7 %) and using up all of respondent’s savings (38.5 %). Other financial challenges less commonly reported included taking on credit card debt (30.9 %), receiving a lower credit rating (20.9 %) and being contacted by a collection agency about owing money for medical bills (19.9 %) (see Table 2).

**Table 2:** Prevalence of medical debt outcomes and associations between guarantor characteristics and medical debt outcomes, 2015 CarePayment sample

Medical Debt Outcome	Descriptive Results	Results of Logistic Regression Models^a^									
	Percent of Respondents	Household Income (Comparison: $80,000 or more)^b^				Amount of debt (Comparison: Less than $2,000)^b^				Service Usage^b^	
		Less than $20,000	$20,000–39,999	$40,000–59,999	$60,000–79,999	$2,000–3,999	$4,000–7,999	$8,000–9,999	$10,000 or more	First-time CP User (Y)	Other medical debt (Y)
*Because of medical bills, in the last two years…*											
Unable to pay for necessities (food, heat, rent)	14.4	8.0**	4.8*	4.4*	x	x	x	x	2.4*	x	2.5*
Used up all your savings	38.5	2.4*	2.3*	2.5*	2.0*	x	x	x	4.4**	1.6*	2.8**
Taken out a mortgage or loan	9.6	x	x	x	x	x	x	x	4.3**	x	2.3*
Taken on credit card debt	30.9	x	x	x	x	x	x	x	2.02*	x	4.2**
Thought about filing for bankruptcy	8.9	x	x	3.2*	x	x	x	3.4*	x	x	3.2*
Had to declare bankruptcy	2.8	x	x	x	x	x	x	x	x	x	x
Received a lower credit rating	20.9	3.1*	2.0*	x	x	x	x	x	x	x	3.0**
Delayed education or career plans	9.6	3.5*	x	x	3.4*	x	x	x	3.8*	2.1*	2.9*
*Because of cost, in the past 12 months…*											
Did not fill a prescription	28.9	x	x	x	x	x	x	x	2.1*	x	3.2**
Skipped a medical test, treatment or follow-up recommended by a doctor	32.9	x	x	x	x	x	x	x	x	1.6*	3.4**
Skipped doses of a prescription medicine or cut pills	24.6	x	2.3*	x	x	x	x	x	2.1*	x	3.1**
Had a medical problem but did not go to a doctor/clinic	30.3	x	1.8*	2.6**	x	x	x	x	x	x	2.9**
Did not see a specialist when you/your doctor thought you needed one	24.0	x	x	x	x	x	1.8*	x	2.2*	x	2.1**
Delayed or skipped preventive care screening	24.6	x	x	x	x	x	x	x	2.1*	1.8*	2.7**
*In the past 12 months…*											
Problems paying/unable to pay medical bills	59.5	3.2**	2.5**	2.5**	x	x	x	x	x	x	2.8**
Had to change way of life significantly in order to pay medical bills	38.7	4.9**	4.4**	2.6*	2.7*	x	x	x	8.7**	x	2.4**
Contacted by a collection agency about owing money for medical bills	19.9	x	x	x	x	x	x	x	x	1.5*	3.1**

^a^Each row represents one logistic regression model with outcome variable representing the odds of the respondent reporting the negative outcome listed in the first column

^b^Cells present Odds Ratios from the full model for significant co-variates only

*p < 0.05; **p < .001; x: *p* > 0.05

As mentioned above, health care access challenges are also common among individuals with medical debt. In the CP guarantor sample, the most commonly reported access challenges within the past 12 months, due to cost, were skipping a medical test, treatment or follow-up recommended by a doctor (32.9 %), having a medical problem but not going to the doctor/clinic (30.3 %) and not filling a prescription due to cost (28.9 %). Less frequently reported challenges included skipping doses of a prescription medicine or cutting pills (24.6 %), delaying or skipping preventive care screening (24.6 %) and not seeing a specialist when their doctor thought they needed one (24.0 %).

### Predictors of negative outcomes

In order to explore the impact of CP on medical debt-related outcomes, a series of regression models was run to explore attributes that were associated with an increased odds of experiencing each of these outcomes. Given the lack of comparison group consisting of non-CP users, comparisons were made between first-time CP guarantors (*n* = 714) and repeat customers (*n* = 272). It was assumed that if CP use is associated with reductions in the negative outcomes, repeat users would be less likely to report the outcomes compared to first time users. We also separated individuals with CP debt only (*n* = 398) from those with both CP debt and other medical debt (*n* = 598) to begin to explore the impact of CP debt separately from other types of debt. In order to explore the impact of these characteristics on the negative outcomes measured, a series of logistic regression models was run, one for each of the negative outcomes (i.e., seventeen models in total). Within each model, both household income and the amount of debt were included as control variables since the literature suggests that these are both powerful predictors of the negative outcomes measured. The results of the models suggest that first-time users of CP were significantly more likely than repeat users to report being contacted by a collection agency about owing money for medical bills (OR = 1.5); using up all of their savings (OR = 1.6); delaying education or career plans (OR = 2.1); skipping a medical test, treatment or follow up due to cost (OR = 1.6); and delaying or skipping preventive care screening (OR = 1.8). The models also suggest that compared to individuals with only CP debt, those with both CP and other medical bills were significantly more likely to report nearly all of the undesirable financial and access outcomes. Consistent with the literature, we found that total amount of debt was significantly associated with many of the financial and access outcomes measured. The results of these models are presented in Table 2.

## Discussion

This research described both the characteristics of individuals who take advantage of CP and the medical debt related outcomes they faced. In general, the guarantor respondents were diverse in terms of education level but were fairly consistent in terms of marital status (63.6 % were married) and employment status (52.4 % working full-time), and the majority did not have any dependents living in their home.

The results suggest that CP use, especially repeat CP use, may be associated with a reduction in many negative outcomes typically found in individuals with medical debt. Our study was not able to explore the mechanisms for this difference; future research should include qualitative and other methods designed to explore both the mechanism and ways to expand it. However, most respondents (71.6 %) reported no previous experience with CP, suggesting that there may be an opportunity to expand the program and/or encourage first time users to become repeat users which may result in greater improvements in these negative outcomes.

In addition, while we found that individuals with only CP debt fared better than those with both CP debt and other medical debt, fully 60 % of our sample had more than one source of medical debt. This suggests that the beneficial impact of CP could be increased by expanding access to the program and/or allowing other medical bills to be rolled into the monthly CP payments.

Moreover, by the fact that they communicate with large groups of patients and families on a monthly basis, programs like CP have an opportunity to promote better understanding of medical debt among the broader population. For example, 25.2 % of CP phone survey respondents did not know the amount of their annual health insurance deductible. This suggests a need for greater health and financial literacy in the area of insurance, and highlights an opportunity for programs like CarePayment to take the lead in promoting greater understanding.

The study had a small number of weaknesses that may have impacted the results. For one, eligibility for CP varies from hospital to hospital and may relate to an individual’s financial status (e.g. non-payment of past debt). This would lead to our sample being more financially sound than other Americans with medical debt. Future analyses comparing the sample to nationally representative samples of individuals with medical debt are planned. In addition, our relatively low response rate may have introduced bias into the sample. Post hoc analyses were conducted, comparing the total amount of CP debt guarantors were paying off between responders and non-responders. The distribution of debt was comparable between the two groups (data not shown), though differences in other domains could lead to our sample not being representative of the larger CP guarantor pool. Data on the full sample were not available for other attributes, so additional analyses were not possible.

## Conclusions

Although the overall rate of medical bill problems and/or debt has declined significantly since 2010, medical debt and the associated financial and access to care challenges remain important concerns, particular among the uninsured, the underinsured, and low- to middle-income households. Given this reality, programs such as CarePayment, which shows promise in reducing many of the financial and access to care problems commonly associated with medical debt, should be included in discussions in the public health, medical care, and payer communities about how to ameliorate the negative consequences of medical debt.

## Abbreviations

ACA, Affordable Care Act; BAA, Business Associates Agreements; BHIS, Biennial Health Insurance Survey; CP, CarePayment; FAP, financial assistance plans; OR, odds ratio; PMR, Pacific Market Research.
